# Improving the Accuracy of Endoscopic Measurements of the Size of Gastrointestinal Lesions by a Guidewire Scale

**DOI:** 10.1155/grp/3466541

**Published:** 2026-02-16

**Authors:** Nanthawat Talalak, Saritphat Orrapin, Sittichock Wattanarochanaporn, Teerawat Sotananan, Thanakorn Likidkarnchanakornkij, Prasit Mahawongkajit

**Affiliations:** ^1^ Department of Surgery, Sakaeo Crown Prince Hospital, Sakaeo, Thailand; ^2^ Department of Surgery, Rajavithi Hospital, Pathum Thani, Thailand, rajavithi.go.th; ^3^ Department of Surgery, Faculty of Medicine, Thammasat University, Pathum Thani, Thailand, tu.ac.th

**Keywords:** endoscopic measurement, esophagogastroduodenoscopy, gastric lesion, guidewire scale

## Abstract

**Objectives:**

Using an endoscope to accurately measure the size of a lesion is crucial for assessing the healing process and determining the treatment strategies for the stage of the cancer. Visual estimation of the lesion size is commonly used, but this is often inaccurate. To address this issue, a guidewire scale was developed to measure lesion size and improve accuracy at a practical level.

**Methods:**

Four endoscopists estimated the size of artificial lesions in the gastric model by visual comparison with the guidewire measurements. The endoscopists used a guidewire scale to determine the size of the lesion and rated their satisfaction after the procedure using a numeral rating scale (NRS).

**Results:**

Fifty‐two patients were recruited and assessed for the size of their lesions using a guidewire scale. Overall, the endoscopists reported good satisfaction levels (NRS ≥ 8), with satisfaction significantly higher in the dyspepsia group (*p* = 0.03). The mean lesion size was 10.7 ± 8.9 mm, with smaller lesions (≤ 1 cm) resulting in higher satisfaction levels. On average, it took 39.4 ± 13.6 s to measure the lesion, with a procedure time of ≤ 30 s resulting in very high satisfaction levels.

**Conclusions:**

The accuracy of endoscopic measurements was improved using the guidewire scale, allowing clinical studies to safely and easily estimate lesion sizes.

## 1. Introduction

Esophagogastroduodenoscopy (EGD) is a commonly used diagnostic and therapeutic procedure in gastroenterology and upper gastrointestinal (GI) surgery [1] and an essential tool for screening precancerous lesions and diagnosing and staging upper GI cancer [2, 3]. The size of the lesion determined during endoscopic surveillance is used to assess the treatment strategies for ulcer healing [4, 5].

Several studies have suggested that the size of an ulcer may be associated with lymph node metastasis in early gastric cancer (EGC) [6, 7]. Endoscopic therapy is a safe and effective treatment for EGC as a minimally invasive procedure that enables the patient to keep their entire stomach and maintain a good quality of life [8]. Accurately measuring the tumor size is crucial for selecting the right candidate for endoscopic resection (ER) of EGC [9]. The 2021 Japanese gastric cancer guidelines suggested that tumors with a diameter of less than or equal to 2 cm require endoscopic mucosal resection (EMR), whereas tumors with a diameter of more than or equal to 2 cm require endoscopic submucosal resection (ESD) [10]. As mentioned earlier, tumor size is a major determinant of any treatment plan.

The significance of accurately measuring the size of a lesion has been previously acknowledged. Traditionally, endoscopists have visually estimated the size of the lesion, but this approach has limitations and can result in significant measurement errors [11]. For this reason, several endoscopic measurement methods have been developed. Initially, the measurement technique involved comparing the lesion with objects of a known size such as a rubber disk or open biopsy forceps according to the Paris endoscopic classification of EGC [12, 13]. However, studies have shown that the open biopsy forceps technique often underestimated the size of the lesion [14, 15]. To address these limitations, other measurement programs such as laser endoscopy, image processing with a grid scale, and three‐dimensional (3D) stereoendoscopy systems have been developed [16–19]. These techniques improved the accuracy of measuring the size of lesions, but they required additional devices that increased the complexity and costs.

To date, no standardized method exists to measure a gastric lesion [20]. Therefore, this study developed a technique that met the following three criteria: (1) simple and easy to implement, (2) cost‐effective, and (3) accurate at a practical level. Our research focused on enhancing the precision of measuring the size of the lesion using a guidewire scale.

## 2. Materials and Methods

### 2.1. Guidewire Scale

**Figure 1:**
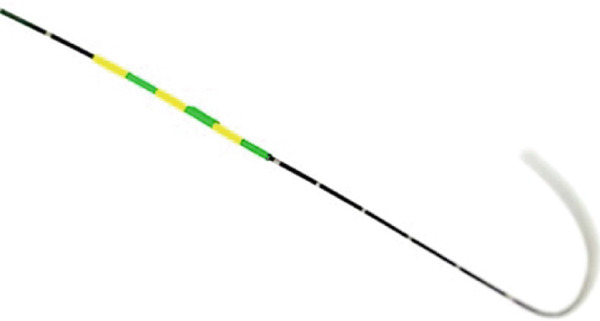
Guidewire scale.

The flabby tip was removed from a guidewire to make it scalable. Green and yellow stickers were applied to the wire (5 mm diameter and 30 mm length) in an alternating pattern. The scale was marked 10 cm from the tip of the guidewire (Figure 1).

### 2.2. Measurement in the Gastric Model

To validate the accuracy of a guidewire scale, artificial gastric ulcers were created in a model box measuring 25 × 35 × 20 cm. The ulcers were made of white modeling clay and ranged in size from 2.5 to 30 mm. Each size had five lesions, making a total of 60 lesions. The endoscope was fixed at a 60° angle, and each lesion was fixed on a mock‐up stomach. The tip of the endoscope was withdrawn at a constant distance of 5 cm from the lesion. Four experienced endoscopists, who had performed over 1000 cases of EGD, visually estimated the size of the 60 lesions randomly. The mean measurement error was then calculated.

After visually estimating the size of 60 artificial ulcers, a single operator conducted the measurements using a guidewire scale. Mean measurement errors were evaluated and compared between visual estimation and the guidewire scale measurements.

### 2.3. Measurement in Clinical Study

This clinical study received approval from the Human Research Ethics Committee at Sakaeo Crown Prince Hospital (S012h/65 ExPD). Before participating in the study, all patients provided written informed consent. The endoscopists used a conventional instrument and technique to perform EGD and then fixed the endoscope to evaluate the entire lesion using a guidewire scale. They observed and determined the endoscopic findings and locations.

To estimate the lesion size using a guidewire scale, the endoscopists recorded the size of the lesion in millimeters, the completion rate of the procedure as a percentage, and the time of the procedure in seconds from the insertion to the removal of the guidewire. The ease of the measuring procedure and endoscopist satisfaction were evaluated using a numeral rating scale (NRS) ranging from 0 to 10, with 0 indicating *dissatisfaction* and 10 indicating *complete satisfaction*.

Following the procedure, the research team asked the patients about symptoms such as nausea, vomiting, cough, and abdominal discomfort.

### 2.4. Statistical Analysis

The results were presented as mean ± standard deviation (SD). Statistical analyses were conducted to compare the diameters of all types of lesions estimated virtually and using a guidewire scale with their actual size. Linear correlations were evaluated by the paired *t*‐test and Pearson’s correlation coefficient (*r*). For nonparametric data or nonbivariate normality, Spearman’s rank correlation coefficient analysis was performed, with the Shapiro–Wilk *W* test and Doornik–Hansen test used to examine the normality of the data. Intraclass correlation coefficient (ICC) analysis was performed to assess agreement and reliability, while the Bland–Altman analysis was used to evaluate measurement concordance between the visual estimation using the guidewire scale and the actual artificial lesion size for Endoscopists 1, 2, 3, and 4. All data were analyzed using Stata/SE 16.0 for Mac (StataCorp, Texas, United States). A *p* value of less than 0.05 was considered statistically significant.

## 3. Results

### 3.1. Visual Estimation Measurement in Gastric Lesion Model

**Figure 2:**
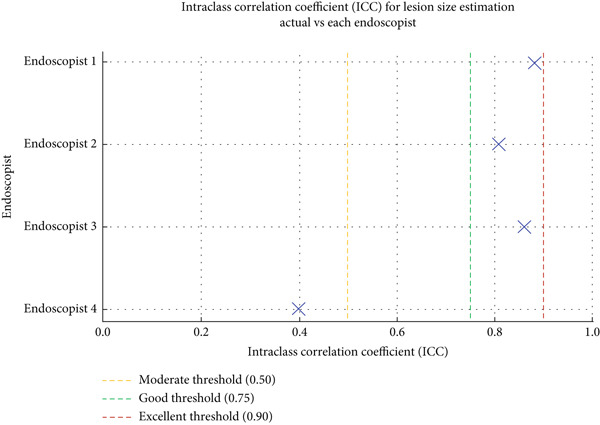
Interclass correlation coefficient (ICC) for lesion size estimation comparing actual artificial lesion size with visual estimation by Endoscopists 1, 2, 3, and 4. Vertical dash lines indicated interpretation thresholds: moderate (0.50), good (0.75), and excellent (0.90) reliability.

Four endoscopists visually estimated the size of 60 artificial ulcers. The measurement errors of Endoscopists 1, 2, and 4 were −0.23 ± 0.36, 0.19 ± 0.46, and 0.83 ± 0.52 mm, respectively (*p* < 0.001), while the measurement error of Endoscopist 3 was −0.05 ± 0.41 mm (*p* = 0.28). The Shapiro–Wilk *W* test indicated a normal distribution, and the Doornik–Hansen test showed nonbivariate normality. The results of Spearman’s rank correlation coefficient showed a very high positive correlation between Endoscopists 1 and 4 (*p* < 0.001; *r* = 0.91 and 0.94) and a highly positive correlation between Endoscopists 2 and 3 (*p* < 0.001; *r* = 0.83 and 0.88). Endoscopists 1, 2, and 3 show strong reliability (ICC > 0.75) when estimating lesion size compared to the actual measurement. Endoscopist 4 demonstrated weaker consistency (ICC = 0.397), suggesting possible measurement bias or variation (Table 1 and Figure 2). The Bland–Altman plots above illustrate mean bias and ± 1.96 SD limits of agreement for each comparison—most differences fall within acceptable limits for Endoscopists 1, 2, and 3, but wider variation is seen with Endoscopist 4 (Figure 3).

**Table 1:** The relationship between visual estimation and actual artificial lesion size by four endoscopists.

**Endoscopists**	**Mean different**	**p** **value**	**Correlation coefficient (Spearman’s rho (** **r**_**s**_ **))**	**p** **value**	**Effect size of correlation**	**Intraclass correlation coefficient (ICC)**	**Upper LoA (+1.96 SD)**	**Lower LoA (−1.96 SD)**
Lesion size Endoscopist 1	−0.23 ± 0.36	< 0.001	0.912	< 0.001	Very highly positive correlation	0.881	0.47	0.93
Lesion size Endoscopist 2	−0.19 ± 0.46	0.001	0.833	< 0.001	Highly positive correlation	0.807	1.10	0.71
Lesion size Endoscopist 3	−0.05 ± 0.41	0.281	0.887	< 0.001	Highly positive correlation	0.859	0.74	0.86
Lesion size Endoscopist 4	−0.83 ± 0.52	< 0.001	0.944	< 0.001	Very highly positive correlation	0.397	1.85	0.19

Figure 3The Bland–Altman analysis demonstrating the agreement between visual estimation by guidewire scale and actual artificial lesion size of Endoscopists (a) 1, (b) 2, (c) 3, and (d) 4.

**Figure:**
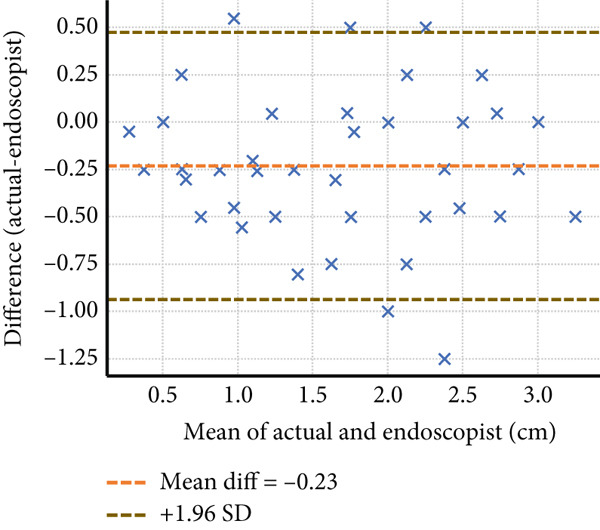
(a)

**Figure:**
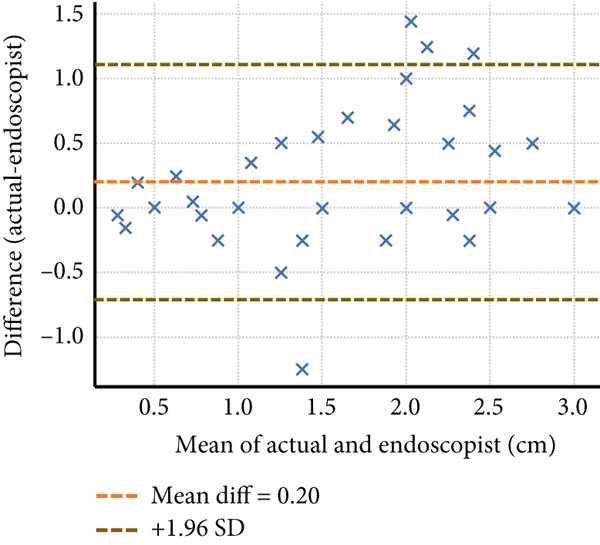
(b)

**Figure:**
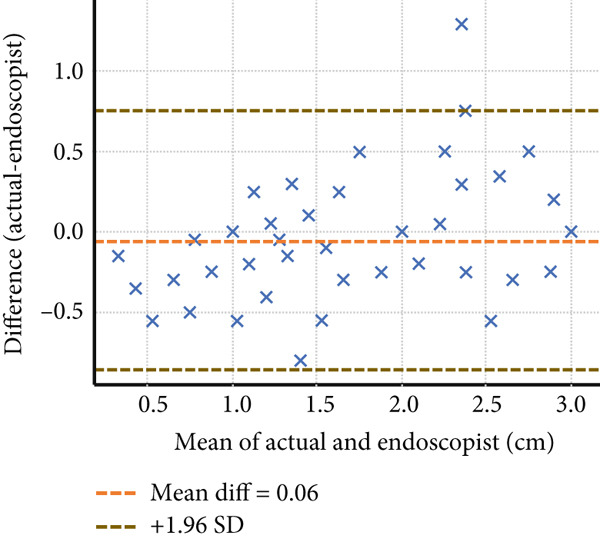
(c)

**Figure:**
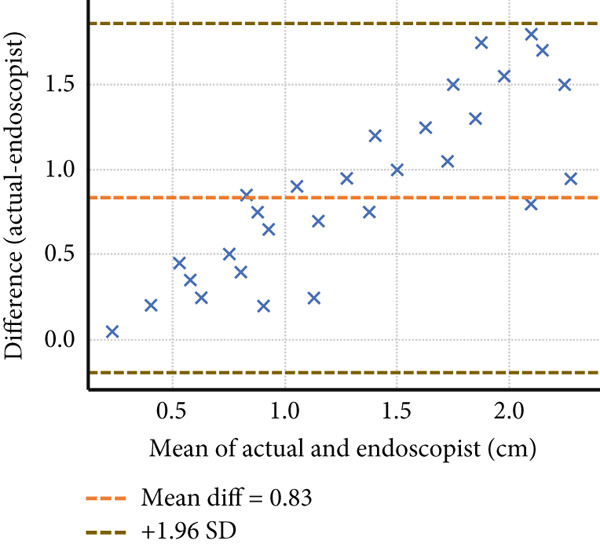
(d)

### 3.2. Guidewire Scale Measurement in Gastric Lesion Model

**Figure 4:**
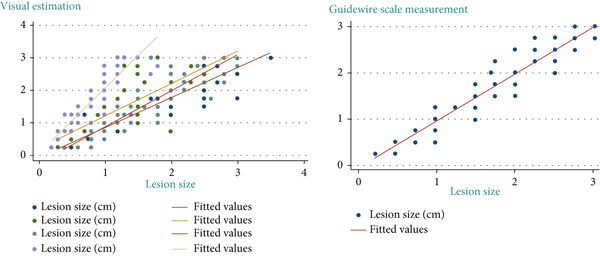
Relationship between visual estimation and actual artificial lesion size of four and relationship between guidewire measurement and actual artificial lesion size.

Sixty artificial gastric lesions were measured using a guidewire scale. The average time for the procedure was 43.73 s, and the measurement error was −0.36 ± 0.25 mm (*p* = 0.26). The data had a normal distribution and bivariate normality using the Shapiro–Wilk *W* test. A very positive correlation was observed using the Doornik–Hansen test (*p* > 0.05; *r* = 0.95). The linear relationship between the actual size and guidewire scale measurement was reported as fair (*k* = 0.382) (Figure 4).

### 3.3. Guidewire Scale Measurement in Clinical Study

Fifty‐two patients were recruited to evaluate the size of their gastric lesions using a guidewire scale (Table 2). All procedures were completed successfully without any complications. The endoscopists reported a good level of satisfaction (NRS ≥ 8) overall, but the satisfaction was significantly higher in the dyspepsia group (*p* = 0.03, CI = 0.93–16.04, OR = 3.87). The mean size of the lesion was 10.7 ± 8.9 mm, and smaller ulcer sizes (size ≤ 1 cm) resulted in higher satisfaction. It took an average of 39.4 ± 13.6 s to measure the lesion, and a procedure time ≤ 30 s was reported as highly satisfactory (Table 3).

**Table 2:** Patient characteristics.

**Characteristics**	**Value**
Gender	
Male, *n* (%)	31 (59.61%)
Female, *n* (%)	21 (40.39%)
Age (years)	61.63 ± 12.82
Body weight (kg)	60.10 ± 8.75
Height (m)	159.56 ± 8.75
BMI	23.56 ± 4.37
Current smoker, *n* (%)	10 (19.23%)
Current alcohol drinker, *n* (%)	10 (18.18%)
Indications for EGD	
Dyspepsia, *n* (%)	12 (21.82%)
Dysphagia, *n* (%)	2 (3.85%)
Upper gastrointestinal hemorrhage, *n* (%)	33 (63.46%)
Anemia, *n* (%)	4 (7.69%)
Prior experience of EGD, *n* (%)	16 (30.76%)

**Table 3:** Endoscopic findings and endoscopist’s satisfaction.

**Endoscopic findings and satisfaction**	**Mean different**	**Good endoscopic satisfaction**	**Poor endoscopic satisfaction**	**p** **value**
Ulcer size (mm)	10.71 ± 8.94	8.12 ± 8.07	14.06 ± 9.05	0.013
Time (s)	39.40 ± 13.69	29.70 ± 3.64	51.91 ± 11.53	< 0.001
Rate of completion, *n* (%)	52 (100%)	—	—	—
The ease of procedure, mean ± SD, 0–10 NRS	8.98 ± 0.99	9.37 ± 0.81	6.8 ± 0.54	< 0.001
Endoscopist satisfaction, mean ± SD, 0–10 NRS	8.63 ± 1.38	9.06 ± 0.92	7.0 ± 0	< 0.001
Location of ulcer				
Cardia, *n* (%)	1 (3.23%)	1 (3.23%)	0 (0%)	0.37
Fundus, *n* (%)	1 (1.92%)	1 (3.23%)	0 (0%)	0.37
Body, *n* (%)	2 (3.85%)	1 (3.23%) 4 (12.90%)	0 (0%) 3	0.37
Antrum, *n* (%)	7 (13.46%)	17 (54.84%)	(12.50%) 18 (75%)	0.96
Pylorus, *n* (%)	33 (63.46%)	6 (20%)	2 (8.33%)	0.12
Duodenum, *n* (%)	9 (17.31%)			0.23

*Note:* NRS ≥ 8 being good satisfaction and NRS < 8 being poor satisfaction.

## 4. Discussion

Accurately measuring the size of a tumor is crucial in identifying suitable candidates and achieving successful outcomes in ER of EGC. Lesion size plays a significant role in risk assessment, treatment planning, procedural safety, and postprocedural surveillance. Despite its importance, accurately measuring the size of GI lesions remains a challenge [11]. Currently, there is no standardized approach for estimating the size of a lesion during an endoscopic procedure. However, a simple way to measure lesion size is by comparison with a known‐sized object such as open biopsy forceps. This method is recommended by the Paris endoscopic classification guideline. To address this issue, this clinical research introduced a novel endoscopic guidewire scale and evaluated its accuracy.

A guidewire scale is a comparison technique used to assess the diameter of a lesion without the need for additional devices or complex instruments. This method relies on a guidewire scale as a standard reference. Our team developed a simple and cost‐effective measurement option for secondary care services that is easy to use and accurate in practice, as the simplest measuring technique available.

Various methods have been proposed to accurately determine the size of GI lesions. Traditionally, lesions were estimated visually in a multicentre. Visual estimation trials were conducted in a gastric model, which showed differences in measurement errors among the four operators. Three endoscopists made significant errors in their visual estimation, while the fourth estimated the size accurately compared to the actual size of the artificial lesion. This observation proved that the visual estimation method relied heavily on the operator.

After conducting guidewire scale measurements in the gastric model, the correlation analysis showed an increase in linear relationship association, while using a guidewire scale technique with a single operator resulted in no significant errors compared to the actual sizes of the lesions. The correlation analysis revealed a very high positive correlation in the guidewire method, while visual estimation varied between individuals. Results suggested that the guidewire method improved accuracy compared to visual estimation.

During the clinical study, each patient underwent an unsedated EGD with a standard endoscope and without additional instruments. The endoscope tip was placed as close as possible to the lesion to allow a thorough evaluation. A guidewire scale was then inserted through a biopsy channel, and the endoscopist manipulated it along the curve of the stomach to assess any gastric lesions under endoscopic view. In practice, it was easier to control the wire in the stomach due to its smaller size and curve compared to the gastric model. The average time taken for the measurement procedure in the stomach was less than in the gastric model, and the endoscopists reported being more comfortable after performing the procedure for 10 cases. In some cases, the procedure took longer due to lower esophageal sphincter (LES) relaxation difficulties. Overall, the endoscopists reported a satisfactory experience with the guidewire scale in practical applications.

This study was limited as the guidewire was only capable of one‐dimensional measurement. However, a prototype of a novel tool was created using a recycled guidewire, with the potential to become a standard commercial instrument in the future. To ensure accuracy, the guidewire measurement should be tested by multiple endoscopists because operator dependency may introduce errors. A larger sample size and multicentre study are needed to render more statistically significant results with a power of at least 80%.

Our guidewire scale improved the accuracy of endoscopic measurements of lesion size. This scale can be used safely and easily to estimate sizes in clinical studies. However, further replication of a guidewire scale measurement in patients is necessary to contribute to the study of endoscopy across multiple centers and with different levels of endoscopist experience.

**Nomenclature**
EGD: esophagogastroduodenoscopy
GI: gastrointestinal
EGC: early gastric cancer
ER: endoscopic resection
EMR: endoscopic mucosal resection
ESD: endoscopic submucosal dissection
cm: centimeter
mm: millimeter
LES: lower esophageal sphincter

## Conflicts of Interest

The authors declare no conflicts of interest.

## Funding

No funding was received for this manuscript.

## Data Availability

The data that support the findings of this study are available from the corresponding author upon reasonable request.
